# Preparation of Highly Uniform Silica Microspheres Recycled from Silicone Rubber and Their Application as Fillers in Epoxy Resin-Based Insulating Materials

**DOI:** 10.3390/ma18245647

**Published:** 2025-12-16

**Authors:** Zhiling Chen, Li Cheng, Wenlong Xu, Ruijin Liao

**Affiliations:** State Key Laboratory of Power Transmission Equipment Technology, School of Electrical Engineering, Chongqing University, Chongqing 400044, China; zhiling_chen@stu.cqu.edu.cn (Z.C.);

**Keywords:** decommissioned composite insulators, silicone rubber recycling, silica microspheres, post-treatment particle size fractionation, insulation performance

## Abstract

**Highlights:**

With the growing number of decommissioned composite insulators from power transmission systems, waste silicone rubber has become an emerging environmental concern. This material poses significant challenges due to its non-degradable nature and the large volumes requiring disposal. This work, proposes a high-value recycling approach that converts end-of-life silicone rubber into highly uniform SiO_2_; microspheres, achieved through a combined process of stepwise pyrolysis and post-fractionation.

**What are the main findings?**

- A stepwise pyrolysis and electrophoretic fractionation route converts waste silicone rubber into uniform SiO_2_ microspheres.
- Stepwise pyrolysis and electrophoretic fractionation enabled particle size control.
- Recycled SiO_2_ achieved comparable dielectric enhancement to commercial fillers.

**What is the implication of the main finding?**

- Provides a high-value, waste-to-resource solution for non-degradable silicone rubber from power systems.
- Demonstrates the technical and economic feasibility of closed-loop recycling in the electrical industry.
- Contributes to sustainable material cycles and greener production in high-voltage insulation.

**Abstract:**

Silicone rubber from decommissioned composite insulators has become one of the major environmental challenges in the power industry due to its non-degradable nature. Therefore, the recycling and reuse of silicone rubber are of great environmental and economic significance. In this work, a method for preparing silica microspheres based on stepwise pyrolysis combined with post-treatment particle size fractionation is proposed. First, highly spherical silica microspheres were obtained by stepwise pyrolysis. Subsequently, glass fiber membrane filtration and aga-rose gel electrophoresis were employed as post-treatment methods to achieve particle size fractionation and enhanced uniformity. The results indicate that the post-treated silica microspheres exhibit high uniformity, high sphericity, and good dispersibility. This method significantly improves the structural uniformity and microscopic characteristics of the microspheres, making them promising high-value fillers for epoxy resin insulation modification. Comparative analysis with commercial nanosilica used as epoxy fillers shows that the recycled and fractionated silica microspheres achieve comparable improvements in breakdown strength and dielectric performance, confirming their potential for recycling and reuse in high-voltage insulation and electronic packaging applications.

## 1. Introduction

Composite insulators are widely used in power systems, particularly in high-voltage and ultra-high-voltage transmission lines [1,2,3,4]. Compared with traditional porcelain or glass insulators, composite insulators offer advantages such as low weight, high mechanical strength, and excellent corrosion resistance, which enable superior performance under harsh environmental conditions and significantly enhance the stability and safety of power transmission systems. From 2024 to 2027, large-scale equipment upgrading in power grids is expected to reach a total investment of 600 billion CNY, with more than 15 million composite insulators already in service. As the primary insulating component, silicone rubber generates approximately 6500 tons of decommissioned waste annually, and this volume is still increasing rapidly.

The improper disposal of non-degradable silicone rubber from composite insulators not only results in material waste for power utilities but also imposes considerable environmental pressure. Therefore, how to recycle and reuse massive quantities of decommissioned silicone rubber in a green, low-carbon, and safe manner—so as to match the growing retirement scale with efficient resource recovery capacity and eventually achieve full life-cycle environmental sustainability—has become a pressing challenge for both the present and the coming years [5,6].

Conventional recycling methods for silicone rubber include mechanical pulverization, acid/alkali depolymerization, biodegradation, and high-temperature calcination. However, these methods suffer from issues such as low economic value, high energy consumption, or secondary pollution. Mechanical treatment only yields low-value rubber powder (approximately 2 CNY/kg). Acid/alkali depolymerization can produce cyclic siloxanes, but the process involves strong corrosiveness and flammable gas emissions, posing environmental and safety risks. Biodegradation requires long processing periods (typically over 30 days), and the market value of the resulting products is less than 50 CNY/kg. Overall, the low added value of recycled products is the core bottleneck restricting the large-scale reuse of waste silicone rubber [7,8,9,10,11]. In 2021, Chongqing University first proposed a stepwise pyrolysis approach to recover silicone rubber into submicron silica microspheres [12], providing a new pathway for high-value recycling. In this process, silicone rubber undergoes low-temperature thermo-oxidative cracking (300–600 °C) in a muffle furnace to generate silicon–oxygen precursors, which then self-assemble into spherical particles at higher temperatures (>600 °C), forming high-sphericity silica microspheres. Compared with traditional methods, this technique avoids toxic by-products, and the product value can reach 500–1000 CNY/kg, demonstrating remarkable economic potential. However, in practical recovery processes, problems such as broad particle size distribution, insufficient uniformity, and relatively low purity remain and require further optimization.

To address these challenges, post-treatment particle size fractionation has become a crucial step to improve the quality of silica microspheres [13,14]. Physical filtration using glass fiber membranes can effectively remove large particle impurities, narrow the particle size distribution, and increase product purity. Moreover, introducing electrophoresis-based separation techniques such as agarose gel electrophoresis enables nanometer-level classification of silica microspheres with different particle sizes, significantly enhancing particle size uniformity [15,16,17,18]. Post-treatment not only improves the uniformity, purity, and sphericity of the microspheres but also enhances their interfacial compatibility and insulation performance when used as fillers in composite materials such as epoxy resin.

In this work, waste silicone rubber is used as the raw material, and a “recovery preparation + post-treatment” technical route is proposed. First, high-sphericity silica microspheres (hereafter referred to as SiO_2_ microspheres) are recovered and prepared from silicone rubber via a stepwise pyrolysis method. Then, post-treatment fractionation techniques, including glass fiber membrane filtration and agarose gel electrophoresis, are systematically investigated to evaluate their effects on the uniformity and purity of the microspheres. Furthermore, the performance differences between recycled and commercial SiO_2_ microspheres in epoxy insulation modification are comparatively analyzed to assess the economic benefits of recycling SiO_2_ microspheres from silicone rubber.

## 2. Methods

### 2.1. Materials and Reagents

SiO_2_ microspheres were recovered and prepared from the silicone rubber, which serves as the main insulating component of naturally decommissioned composite insulators. Bisphenol-A type epoxy resin E-51 was selected as the matrix for composite preparation. This resin is chosen due to its excellent comprehensive performance in the field of electrical insulation, its mature processability, and its status as a widely used benchmark for comparison. Methyl tetrahydrophthalic anhydride was used as the curing agent, dimethyl phthalate as the toughening agent, and 1,2-dimethylimidazole as the accelerator. All materials and reagents were purchased from Aladdin Biochemical Technology Co., Ltd. (Shanghai, China). Commercial nanosilica used for performance comparison was provided by NanoMicro Technology Co., Ltd. (Suzhou, China), with a particle size of 100 nm and a purity higher than 99.9%. The 50× TAE electrophoresis buffer was obtained from Solarbio (Beijing, China) (Cat. No. T1060), and agarose was purchased from Biowest (Shanghai, China) (Cat. No. YZQZT).

### 2.2. Chemical Mechanism of SiO_2_ Formation from Silicone Rubber

**Figure 1:**
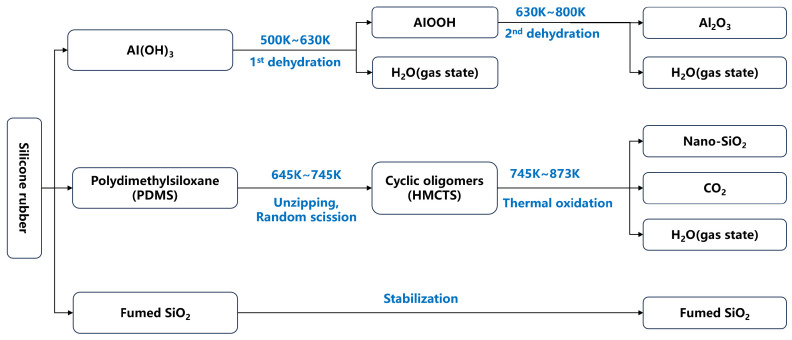
The thermal decomposition process of silicone rubber in retired composite insulators and the recovered products [12].

Previous work revealed that the thermal decomposition of silicone rubber from retired composite insulators in air (illustrated in Figure 1) involves two main stages [12]. The process commences with the dehydration of aluminum hydroxide to alumina, concurrent with the depolymerization of polydimethylsiloxane (PDMS) into volatile cyclic siloxanes, predominantly hexamethyl-cyclotrisiloxane (HMCTS). Subsequently, the HMCTS vapor reacts with oxygen through thermal oxidation to form SiO_2_. Under the quasi-closed conditions of the reactor, this generates a highly supersaturated environment, precipitating SiO_2_ primary particles. These fumed SiO_2_ particles are thermally stable at 1273 K, given their 1650 K melting point.

The conversion of silicone rubber into SiO_2_ microspheres during stepwise pyrolysis involves a sequence of thermal degradation, oxidation, and high-temperature self-assembly reactions. Silicone rubber primarily consists of a −[Si(CH_3_)_2_−O]ₙ− polysiloxane backbone. Under thermo-oxidative cracking, Si–C and Si–O bonds undergo controlled cleavage and oxidation, yielding volatile organic species and silicon-oxygen intermediates that subsequently transform into SiO_2_. The chemical reaction equation is shown as Equation (1):

(1) $${\left[-\mathrm{Si}{\left({\mathrm{CH}}_{3}\right)}_{2}O-\right]}_{n}+4nO_{2}\overset{¦645\sim 873K}{\to }n{\mathrm{SiO}}_{2}+2n{\mathrm{CO}}_{2}+3nH_{2}O$$

### 2.3. Experimental Procedure

**Figure 2:**
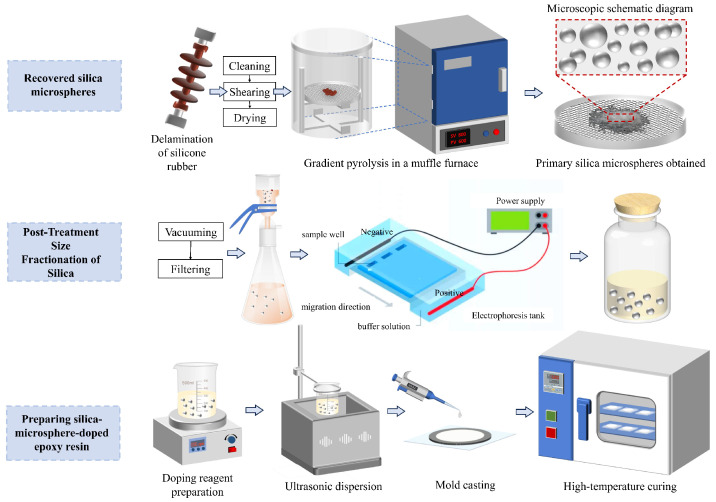
Process for recycling and reusing SiO_2_ microspheres from retired composite insulator silicone rubber.

The overall preparation route for SiO_2_/epoxy resin-based composite materials using recycled SiO_2_ microspheres is illustrated in Figure 2. SiO_2_ microspheres with four different particle sizes (50 nm, 100 nm, 200 nm, and 500 nm) were obtained from silicone rubber via stepwise pyrolysis followed by post-treatment fractionation.

#### 2.3.1. Pretreatment

The silicone rubber sheds of decommissioned composite insulators were first cleaned to remove dust and surface contaminants. The silicone rubber sections were then mechanically cut and dried to obtain fine granulated raw material, improving the efficiency of subsequent recovery.

#### 2.3.2. Stepwise Pyrolysis

After pretreatment, the cleaned silicone rubber particles were placed in a reaction chamber for stepwise pyrolysis. The heating rate of the muffle furnace was set to 20 K/min, and the temperature was gradually increased to the target calcination temperature (300/400/500/600 °C, etc.). The system was held at each stage for 30–240 min. Upon completion, rapid cooling was applied, and the white powder carried by the exhaust gas—identified as SiO_2_ microspheres—was collected. By adjusting the temperature and holding time, the particle size of the recovered microspheres could be controlled: higher temperature and longer residence time resulted in smaller particle sizes.

#### 2.3.3. Glass Fiber Membrane Filtration

For the post-treatment of the recovered SiO_2_ microspheres, the powder was first dispersed ultrasonically in anhydrous ethanol to prevent agglomeration. To further enhance dispersion stability, a silane coupling agent, deionized water, and anhydrous ethanol were mixed in a volume ratio of 1:1:18, allowed to undergo hydrolysis, and then introduced into the SiO_2_ suspension.

Filtration membranes with pore sizes of 0.3 μm and 0.5 μm were used. Under pressure-driven conditions, the SiO_2_ microsphere suspension in deionized water passed through the membranes, allowing the removal of oversized particles and residual impurities. Ultrasonic-assisted dispersion and controlled temperature filtration were adopted to avoid membrane clogging and secondary agglomeration. Through gradient filtration, a sample with a median particle size of approximately 100 nm and a narrowed distribution range was obtained, serving as a pre-fractionated intermediate for subsequent fine separation.

#### 2.3.4. Agarose Gel Electrophoresis-Based Fractionation

To further improve particle size uniformity, agarose gel electrophoresis was employed to fractionate the pre-filtered SiO_2_ microspheres. The agarose gel concentration was set to 0.5 wt%, and 1 × TAE buffer solution was used as the electrophoretic medium. Under a horizontal electric field, SiO_2_ microspheres exhibited differential migration behaviors depending on particle size: smaller particles migrated faster through the gel network, while larger ones were hindered, enabling spatial separation at different migration distances. The applied voltage was controlled within 80–120 V, and the electrophoresis duration was set to 40–60 min. After electrophoresis, the gel was imaged to visualize the fractionation profile, and distinct gel sections were excised. Particles were recovered through water rinsing and ultrasonic dispersion, yielding SiO_2_ microspheres with a nearly monomodal particle size distribution.

#### 2.3.5. Preparation of SiO_2_/EP Composites

The post-treated SiO_2_ microspheres were subjected to surface modification by mixing in solution, heating at 120 °C under continuous stirring and reflux for 10–24 h. The resulting suspension was vacuum dried at 60 °C, and the dried microspheres were ball-milled for 12 h to further reduce agglomeration [19,20].

The SiO_2_ microspheres were then added into the epoxy resin and stirred at 50 °C for 1 h. The curing agent, toughening agent, and accelerator were added sequentially according to the ratio of epoxy resin–curing agent–toughening agent–accelerator = 100:80:10:1. Alternating stirring and ultrasonic dispersion were performed at 50 °C to ensure uniform dispersion.

The mixture was poured into metal molds, degassed under vacuum, and cured thermally under the same curing schedule. After cooling and demolding, circular disc samples were obtained with a diameter of 47 mm and a thickness of 1 mm. The volume fractions of SiO_2_ in the SiO_2_/EP composites were set to 2%, 5%, 8%, 10%, 15%, and 20%. Samples without post-treatment fractionation of SiO_2_ were used as control groups.

### 2.4. Characterization Techniques

To comprehensively evaluate the morphology, particle size distribution, and insulation performance enhancement of recycled SiO_2_ microspheres in epoxy resin, multiple characterization techniques were employed, including scanning electron microscopy (SEM), dynamic light scattering (DLS), breakdown strength testing, and dielectric property measurements.

#### 2.4.1. Microstructural Surface Morphology Analysis

A high-resolution scanning electron microscope (SEM; SU8600, Hitachi High-Tech, Tokyo, Japan) equipped with an energy-dispersive X-ray spectroscopy (EDS) detector (Oxford Instruments, Abingdon, UK) was used to characterize the surface morphology of the recycled SiO_2_ microspheres and the SiO_2_/EP composites. The accelerating voltage was set to 10 kV, and both surface topology and elemental distribution mapping were performed.

#### 2.4.2. Particle Size Distribution Analysis (DLS)

Dynamic light scattering (DLS) was used to analyze the particle size distribution of the recycled SiO_2_ microspheres before and after post-treatment. The particle size distribution was characterized using D10, D50, and D90 (Corresponding to the diameters below which 10%, 50%, and 90% of the total particle volume resides.) to quantitatively describe the distribution boundaries:

- D10 represents the particle size below which 10% of the particles are contained, reflecting the lower boundary of fine particles in the sample.
- D50, also known as the median diameter, indicates the particle size below which 50% of the particles are distributed and serves as a representative characteristic diameter.
- D90 refers to the particle size below which 90% of the particles fall, typically used to describe the upper boundary of coarse particles.

#### 2.4.3. Gel Imaging System

A gel imaging system equipped with a high-resolution CCD camera, multi-wavelength light sources (UV, blue light, and white light), and dedicated optical filters was used to visualize and analyze the agarose gel electrophoresis results. The marker-stained SiO_2_ microspheres were imaged to observe their migration profiles and separation performance during electrophoretic fractionation.

#### 2.4.4. Breakdown Voltage Test

A CS2674C withstand voltage tester was used for breakdown voltage measurements. The power supply range was 0–100 kV, and both the upper and lower electrodes had a diameter of 25 mm. The electrodes and SiO_2_/EP composite specimens were immersed in insulating oil, and the voltage was increased at a rate of 1 kV/s. Breakdown voltage testing was conducted to evaluate the electrical insulation performance of the composites. By comparing breakdown voltages under different SiO_2_ particle sizes and filling ratios, the enhancement effect of SiO_2_ microspheres on epoxy insulation properties was assessed directly.

#### 2.4.5. Dielectric Property Measurement

A broadband dielectric impedance analyzer (Agilent 4294A, Keysight Technologies, Santa Rosa, CA, USA) was used to measure the dielectric constant and dielectric loss of the SiO_2_/EP composites. The frequency range during testing was 0.1 Hz–1 MHz, and all measurements were conducted at room temperature. Based on the testing results, the influence of particle size, filling concentration, and size uniformity of SiO_2_ microspheres on the dielectric behavior of the composites was systematically analyzed.

## 3. Results and Discussion

### 3.1. Microstructural Characterization of Recycled SiO_2_ Microspheres

**Figure 3:**
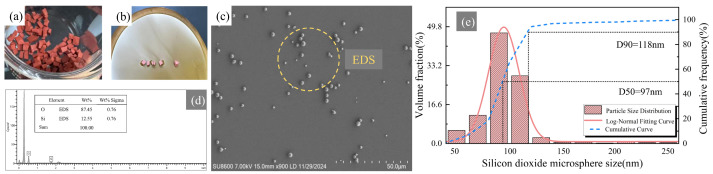
Microscopic characterization of SiO_2_ microspheres—Before post-treatment (**a**) Silicone rubber from retired insulators (**b**) SiO_2_ microspheres obtained through recycling (white powder) (**c**) SEM characterization (**d**) Particle size distribution (**e**) EDS component analysis.

Figure 3 presents the microstructural characterization of SiO_2_ microspheres recovered from the silicone rubber of decommissioned composite insulators under a holding temperature of 873 K for 240 min. The EDS elemental spectrum shown in Figure 3d reveals the elemental composition of the recovered product. The atomic ratio of silicon (Si) and oxygen (O) in the analyzed region is consistent with the theoretical stoichiometric ratio of SiO_2_, confirming that the recovered particles are fully converted silica microspheres.

As shown in Figure 3c, the as-prepared SiO_2_ microspheres (without agarose gel electrophoresis post-treatment) exhibit high sphericity, good dispersion, and relatively smooth surfaces. After multiple-step filtration through glass fiber membranes, large-sized particles (>1000 nm) and residual impurities generated during pyrolysis were effectively removed. However, the particle size distribution, as shown in Figure 3e, remained relatively broad, ranging from 50 to 150 nm, with a D50 of 97 nm and a D90 of 118 nm.

**Figure 4:**
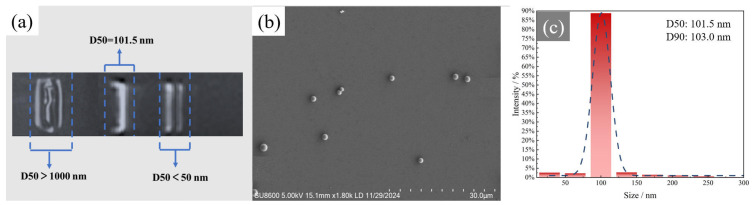
Microscopic characterization of SiO_2_ microspheres—Post-treatment after gel electrophoresis (**a**) Gel electrophoresis imaging result (**b**) SEM characterization (**c**) Particle size distribution.

To further narrow the size distribution, electrophoretic fractionation was conducted based on the difference in surface charge and electrophoretic mobility of SiO_2_ microspheres with different particle sizes. As illustrated in Figure 4a, by precisely controlling the applied electric field and migration time, microspheres with sizes significantly larger than 100 nm remained at the initial loading position, while particles with sizes around 100 nm and 50 nm migrated to different distances within the same electrophoretic period. This enabled high-precision nanometer-scale separation of SiO_2_ microspheres. As shown in Figure 4b,c, after agarose gel electrophoresis treatment, SiO_2_ microspheres with extremely high size uniformity were successfully obtained, achieving a D50 of 101.5 nm and a D90 of 103 nm. Compared with the pre-treated samples, the particle size distribution became significantly narrower, indicating the effectiveness of the post-treatment fractionation.

**Figure 5:**
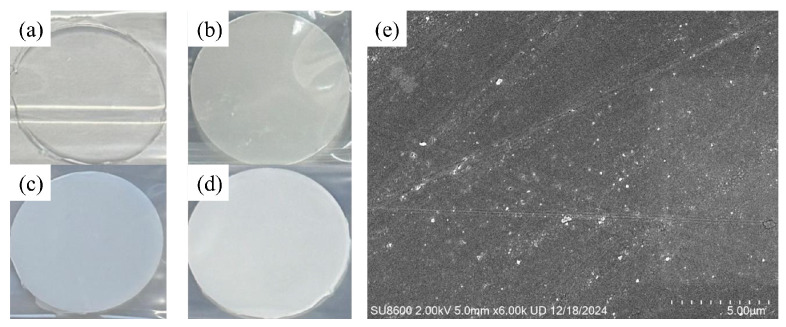
Microscopic characterization of SiO_2_/EP composite materials: (**a**) Pure EP sample; (**b**) 2% SiO_2_/EP sample; (**c**) 5% SiO_2_/EP sample; (**d**) 10% SiO_2_/EP sample; (**e**) 10% SiO_2_/EP sample SEM characterization.

Representative samples of SiO_2_/EP composites with different filling ratios are shown in Figure 5a–d, where the recycled SiO_2_ microspheres used had a nominal particle size of 100 nm. The fracture surface of the composite containing 10 vol% SiO_2_ was sputter-coated with gold and examined using SEM, and the microstructure is presented in Figure 5e. It can be observed that, after dispersion and post-treatment, no obvious particle agglomeration, stacking, or interconnection occurred. Most of the SiO_2_ microspheres were uniformly embedded in the epoxy matrix with a monodispersed spherical morphology.

### 3.2. Breakdown Strength of SiO_2_/EP Composites

For the statistical analysis of breakdown strength in SiO_2_/EP composites, the breakdown field strength corresponding to a cumulative failure probability of 63.2% is typically defined as the characteristic breakdown strength. This value is derived based on the two-parameter Weibull distribution, as expressed in Equation (2):

(2) $$P_{E}=1-\exp\left[-{\left(\frac{E}{E_{0}}\right)}^{\beta }\right]$$

where *E* is the electric field strength at breakdown, *P_E_* is the cumulative probability of breakdown at field *E*, *E*_0_ represents the characteristic breakdown strength corresponding to *P_E_* = 63.2% (1 − e^−1^), and *β* is the shape parameter.

**Figure 6:**
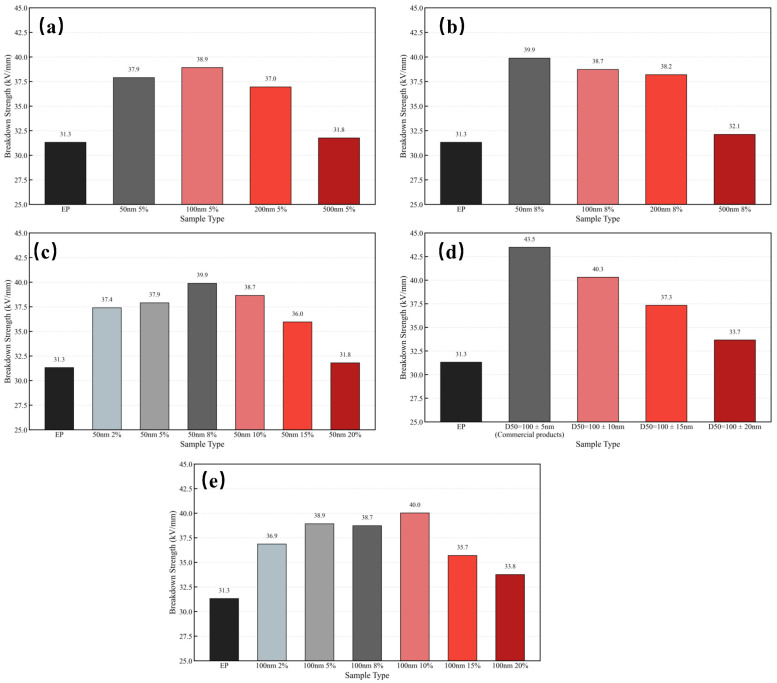
Comparison of breakdown strength of SiO_2_/EP composites. (**a**) Breakdown strength of epoxy resin filled with 5 vol% SiO_2_ microspheres of different particle sizes. (**b**) Breakdown strength of epoxy resin filled with 8 vol% SiO_2_ microspheres of different particle sizes. (**c**) Breakdown strength of epoxy resin filled with SiO_2_ microspheres with different particle size uniformities. (**d**) Breakdown strength of epoxy resin filled with different concentrations of 50 nm SiO_2_ microspheres. (**e**) Breakdown strength of epoxy resin filled with different concentrations of 100 nm SiO_2_ microspheres.

Figure 6 compares the breakdown strength of SiO_2_/EP composites with different particle sizes, loading levels, and particle size uniformity. As shown, the pure epoxy resin (EP) exhibits a breakdown strength of 31.3 kV/mm. Upon the incorporation of recycled SiO_2_ microspheres under various conditions, the breakdown strength of the composites increased significantly. When SiO_2_ microspheres with a particle size of 100 nm, a uniformity higher than 90%, and a filling fraction of 10 vol% were incorporated, the breakdown strength reached a maximum of 40 kV/mm.

From Figure 6a,b, it can be observed that, under the same filling ratio, SiO_2_ microspheres with particle sizes of 50 nm and 100 nm exhibit a stronger enhancement effect, with the maximum increase reaching up to 27.5%. This is attributed to the nano-interface effect, where smaller particles provide a larger specific surface area. Under the same loading, a larger interfacial region is formed between the SiO_2_ microspheres and the epoxy matrix, enabling more efficient trapping of charge carriers and suppressing space charge accumulation. As a result, the local electric field distribution becomes more uniform, reducing field distortion and delaying breakdown initiation.

As shown in Figure 6d,e, when SiO_2_ microspheres with the same particle size were added, the breakdown strength of the composites increased initially and then decreased with increasing filler concentration. A peak enhancement was achieved at 8–10 vol% loading, indicating that this concentration range facilitates the formation of a continuous interfacial network with optimized particle dispersion. This network maximizes charge trapping efficiency. However, when the filler content becomes excessive, inevitable particle agglomeration introduces voids and gas-filled gaps, leading to localized partial discharge and premature breakdown.

Figure 6c further shows that particle size uniformity has a noticeable influence on the breakdown strength of SiO_2_/EP composites. A higher uniformity results in a more pronounced enhancement effect.

Compared with commercial electronic-grade SiO_2_ fillers (Figure 6c and Table 1), the post-treated recycled SiO_2_ microspheres in this study achieved comparable breakdown performance after particle size refinement, demonstrating their potential as high-value functional fillers for electronic encapsulation and high-voltage insulation applications.

### 3.3. Dielectric Properties of SiO_2_/EP Composites

**Figure 7:**
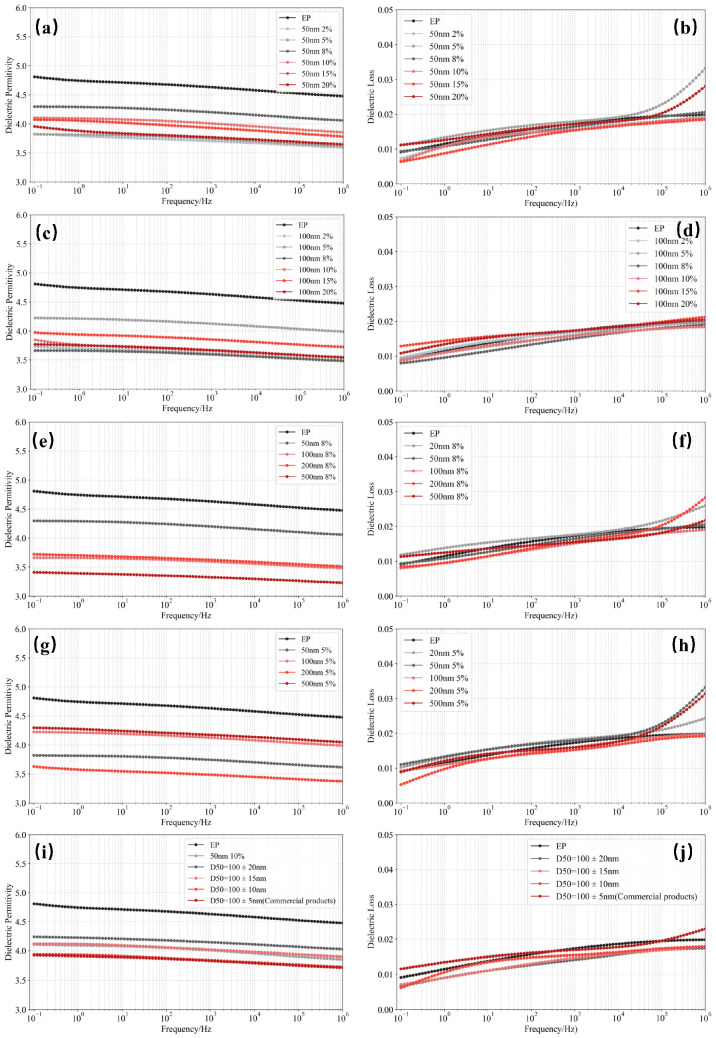
Frequency-dependent dielectric constant and dielectric loss of SiO_2_/EP composites. (**a**) Dielectric constant of epoxy resin filled with different concentrations of 50 nm SiO_2_ microspheres. (**b**) Dielectric loss of epoxy resin filled with different concentrations of 50 nm SiO_2_ microspheres. (**c**) Dielectric constant of epoxy resin filled with different concentrations of 100 nm SiO_2_ microspheres. (**d**) Dielectric loss of epoxy resin filled with different concentrations of 100 nm SiO_2_ microspheres. (**e**) Dielectric constant of epoxy resin filled with 8 vol% SiO_2_ microspheres of different particle sizes. (**f**) Dielectric loss of epoxy resin filled with 8 vol% SiO_2_ microspheres of different particle sizes. (**g**) Dielectric constant of epoxy resin filled with 5 vol% SiO_2_ microspheres of different particle sizes. (**h**) Dielectric loss of epoxy resin filled with 5 vol% SiO_2_ microspheres of different particle sizes. (**i**) Dielectric constant of epoxy resin filled with SiO_2_ microspheres of different particle size uniformities. (**j**) Dielectric loss of epoxy resin filled with SiO_2_ microspheres of different particle size uniformities.

The variation in dielectric constant of SiO_2_/EP composites with different filler sizes and loading fractions as a function of frequency is shown in Figure 7. Overall, the dielectric constant of all samples decreases gradually with increasing electric field frequency, which can be attributed to the inability of dipolar polarization within the material to keep up with the rapid oscillation of the external field at high frequencies.

As shown in Figure 7a,c, compared with pure epoxy resin (EP), the dielectric constant of the epoxy matrix decreases after the incorporation of recycled SiO_2_ microspheres at the same frequency. From Figure 7e,g, it is evident that composites with different SiO_2_ particle sizes exhibit a similar decreasing trend in dielectric constant, and Figure 7i further indicates that a higher particle size uniformity leads to a more pronounced reduction in dielectric constant. These results demonstrate that the incorporation of SiO_2_ microspheres induces an overall decrease in the dielectric constant of the composites. By optimizing the particle size, loading content, and uniformity of SiO_2_ fillers, the dielectric constant reduction performance of recycled SiO_2_ approaches that of commercial high-purity silica (Figure 7i,j and Table 1), suggesting that silica recovered from decommissioned silicone rubber can meet the requirements for high-insulation-performance applications.

The dielectric loss of SiO_2_/EP composites with different SiO_2_ sizes and loading levels as a function of frequency is also shown in Figure 7. As observed in Figure 7b,d,f,g,k, the dielectric loss of SiO_2_/EP composites fluctuates slightly with varying particle size and filler concentration compared to pure epoxy. Although the dielectric loss shows a general upward trend with increasing frequency, and increases more sharply above 100 kHz for some samples, the magnitude of variation remains limited.

This behavior is mainly due to the accelerated motion of polar molecular segments under high-frequency excitation, where increased interfacial friction generates additional heat, leading to an increase in dielectric loss. However, the introduction of SiO_2_ microspheres promotes the formation of an interpenetrating polymer network structure with the epoxy matrix, which suppresses carrier mobility to a certain extent. As a result, the increase in dielectric loss remains moderate, indicating that the SiO_2_/EP composites can meet the operational requirements of high-frequency electronic insulation applications.

To further demonstrate the feasibility of this method, a preliminary assessment of the production cost was conducted. The raw materials used were retired and discarded silicone rubber from the power grid. The material cost was low, so the main cost for production came from the electricity bill generated during the fixed-value heating process in the muffle furnace (a preliminary estimate did not take into account the labor cost, transportation cost, and depreciation cost of the production equipment).

The costs required during the production preparation process are:

(3) $$f=a\cdot W=a\cdot Pt=a\cdot 4\left(t_{1}+t_{2}\right)=4\left(\frac{\theta -\theta_{0}}{60\beta }+t_{2}\right)$$

In the formula: *a* represents the electricity price during the industrial normal period, which is 0.725 CNY/(kW·h); *W* represents the energy consumption of the equipment; *P* represents the power of the equipment, and the power of the Muffle Furnace used in this article is 4 kW; *t* represents the working time of the equipment; *t*_1_ represents the heating-up time; *t*_2_ represents the constant-temperature time; *θ* represents the maximum heating temperature; *θ*_0_ represents the room temperature, which is 293 K; *β* represents the heating-up rate, which is 10 K/min.

It can be calculated that under the conditions of 973K and constant temperature for 2 h, the pyrolysis of 1 kg of waste silicone rubber umbrella skirt results in a production cost of approximately 56.25 yuan for 1 kg of nano-silicon dioxide.

## 4. Conclusions

Recovering and valorizing the non-degradable silicone rubber from decommissioned composite insulators has become one of the key pathways toward achieving green sustainability and enhancing the life-cycle environmental performance of new energy transmission systems. In this study:

1. Combined recovery + post-treatment route—By integrating stepwise pyrolysis with agarose gel electrophoresis, high-uniformity silica (SiO_2_) microspheres were successfully recovered from decommissioned composite insulator silicone rubber. The resulting microspheres exhibited regular smooth spherical morphology, achieved a uniformity of up to 90%, and their diameters were controllably tuned within the 50–500 nm range.
2. Insulation performance enhancement in epoxy composites—The recycled SiO_2_ microspheres were incorporated into epoxy resin to prepare SiO_2_/EP composites with improved insulating properties. When the filler loading was optimized to 8–10 vol% and the particle size was either 50 nm or 100 nm, the breakdown strength increased from 31.3 kV/mm to 40 kV/mm, a rise of 27.5%. At the same time, at a given frequency, both dielectric constant and dielectric loss were reduced; the dielectric constant decreased by 8–21%, while the dielectric loss experienced only a modest increase at high frequencies.
3. Comparable performance to commercial high-precision silica—Through comparative analysis of recycled and commercial nanosilica fillers in epoxy resin, the recycled SiO_2_ microspheres from silicone rubber demonstrated essentially equivalent capability in enhancing breakdown strength and improving dielectric properties. This confirms the feasibility of achieving high-value, economically beneficial recycling of silicone rubber from retired composite insulators.

In summary, the proposed “recovery + post-treatment” strategy offers a viable route for converting decommissioned silicone rubber into functional high-performance SiO_2_ fillers, thereby contributing to sustainable material reuse, value recovery, and cleaner production in the field of high-voltage insulation and electronic packaging.

## Figures and Tables

**Table 1:** Comparison between recycled SiO_2_ microspheres and commercial nanosilica fillers in epoxy composites.

Property	Recycled SiO_2_ (This Work)	Commercial SiO_2_ (100 nm)	Improvement/Comment
Particle size	50–500 nm (controlled)	100 ± 5 nm	Comparable
Uniformity (D90–D10)/D50	<5% after electrophoresis	8–12%	Recycled microspheres have higher monodispersity
Sphericity	High	High	Similar
Breakdown strength (kV/mm)	40 kV/mm at 10 vol%	41–42 kV/mm	Nearly identical
Dielectric constant reduction	8–21%	10–23%	Comparable
Dielectric loss	stable	stable	Similar
Production cost	50 CNY/kg	1500–500,000 CNY/kg	Recycled microspheres are 30–10,000× cheaper

## Data Availability

The data presented in this study are available on request from the corresponding author due to the ongoing nature of the research project and confidentiality agreements, the data supporting the findings of this study are not currently publicly available.

## References

[1] Cao H., Dong R., Zhang X., Qiu W., Qin G., Zhuang Q., Jia Z., Zhang Y., Xi Z., Zhang Z. (2019). Resource Treatment and Green Recycling Application of Decommissioned Composite Insulators in Power Grids.

[2] Liu H., Hu R., Liu Y., Li L., Liu J. (2024). Feasibility study on overall recycling and reuse of 220 kV decommissioned composite insulator core rods. Trans. China Electrotechnol. Soc..

[3] Li H., Liu D., Yao D. (2021). Analysis and prospect of China’s power system development for carbon peak and carbon neutrality targets. Proc. CSEE.

[4] Ma Z., Zhang H., Zhao H., Wang M., Sun Y., Sun K. (2022). New mission and challenges of distribution systems for carbon peak and carbon neutrality. Proc. CSEE.

[5] Lin H., Dong W., Lie J., Yang M., Cui M., Yang D., Yu Q., Liang Y. (2019). Study on the resource utilization of decommissioned composite insulators. New Chem. Mater..

[6] Liang X., Gao Y., Li S. (2016). Rapid development of silicone rubber composite insulators in China. High Volt. Eng..

[7] Hussain S., Zhao Z., Song Y., Zhang C. (2023). Effect of SiO Surface Modification on the Filler-Reinforced Interfaces in SiO-Filled Functional Styrene Butadiene Rubber Composites. J. Appl. Polym. Sci..

[8] Zhang S., Tang N., Cao L., Yin X., Yu J., Ding B. (2016). Highly Integrated Polysulfone/Polyacrylonitrile/Polyamide-6 Air Filter for Multilevel Physical Sieving Airborne Particles. ACS Appl. Mater. Interfaces.

[9] Yang S., Liu Y., Zhou D. (2022). Monomer Recovery and Nano-Silica Separation From Biodegraded Waste Silicone Rubber Shed of Composite Insulator. Front. Mater..

[10] Sabirova A., Florica C.F., Pisig F., Syed A., Buttner U., Li X., Nunes S.P. (2022). Nanoporous Membrane Fabrication by Nanoimprint Lithography for Nanoparticle Sieving. Nanoscale Adv..

[11] Akbulut O., Mace C.R., Martinez R.V., Kumar A.A., Nie Z., Patton M.R., Whitesides G.M. (2012). Separation of Nanoparticles in Aqueous Multiphase Systems through Centrifugation. Nano Lett..

[12] Chen R., Cheng L., Liu Y., Yu L., Liao R., Wang T. (2022). Synthesis of High-purity Mesoporous Nanosilica Microspheres from Retired Composite Insulators Based on Orthogonal Experiment. High Volt..

[13] Hendrix D., Wille K. (2023). Stabilizing Dispersed Colloidal Nanosilica Exposed to an Ultra-High Performance Concrete Environment. Constr. Build. Mater..

[14] Tang X., Sun A., Chu C., Yu M., Ma S., Cheng Y., Guo J., Xu G. (2017). A Novel Silica Nanowire-Silica Composite Aerogels Dried at Ambient Pressure. Mater. Des..

[15] Shojaei M.R., Pircheraghi G., Alinoori A. (2022). Sustainable SBR/Silica Nanocomposites Prepared Using High-Quality Recycled Nanosilica from Lead-Acid Battery Separators. J. Clean. Prod..

[16] Wang R., Xie C., Luo S., Gou B., Xu H., Zeng L. (2019). The Influence Mechanism of Nanoparticles on the Dielectric Properties of Epoxy Resin. RSC Adv..

[17] Yang G., Cui J., Ohki Y., Wang D., Li Y., Tao K. (2018). Dielectric and Relaxation Properties of Composites of Epoxy Resin and Hyperbranched-Polyester-Treated Nanosilica. RSC Adv..

[18] Xie Q., Cheng Y., Chen S., Wu G., Wang Z., Jia Z. (2017). Dielectric and Thermal Properties of Epoxy Resins with TiO_2_ Nanowires. J. Mater. Sci. Mater. Electron..

[19] Zhang S., Wang H., Cheng L., Fang W., Qiu Y., Yang L., Liao R. (2025). Study on the Non-Destructive Evaluation of Overall Particle Dispersion within Nanocomposites by Nonlinear Ultrasonic and Its Applicability. Powder Technol..

[20] Dong H., Liu Y., Cao Y., Wu J., Zhang S., Zhang X., Cheng L. (2021). Terahertz-Based Method for Accurate Characterization of Early Water Absorption Properties of Epoxy Resins and Rapid Detection of Water Absorption. Polymers.

